# Evolution of the human hip. Part 2: muscling the double extension

**DOI:** 10.1093/jhps/hnu014

**Published:** 2014-10-28

**Authors:** Tom Hogervorst, Evie E. Vereecke

**Affiliations:** 1. Haga Hospital, Sportlaan 600, 2566MJ The Hague, Netherlands; 2. Department of Development & Regeneration @ Kulak, KU Leuven, Etienne Sabbelaan 53, 8500 Kortrijk, Belgium

## Abstract

Part 1 of this article outlined the extensive osseous adaptations around the hip that occurred in the development of a habitual bipedal gait in modern humans. The shortest summary of these osseous changes is ‘double extension’, i.e. extension of both the hip joint and the lumbar spine. Not surprisingly, these osseous changes went hand in hand with major muscular changes. The primary changes that accompanied the double extension were changes in relative muscle volume for the quadriceps, gluteus maximus and hamstrings, changes in moment arms for the iliopsoas, gluteus maximus and hamstrings, a change in function for the gluteus medius and minimus, while the functional anatomy of the adductors and hip rotators changed only slightly. The effect of these osseous and muscular changes was improved energy efficiency of human bipedal walking and (long distance) running. However, this occurred at the expense of maximum power, characteristic for activities such as tree climbing (in the apes), but equally so for sprinting. Recognizing these changes and their consequences may help us better understand and treat soft-tissue disorders around the hip.

## INTRODUCTION

No mammal has a habitual extended hip joint position like humans do. Other mammals, including the non-human apes (i.e. gibbon, chimpanzee, bonobo, gorilla and orangutan), have a ‘mid-flex’ hip position as their default. In the preceding section of this two-part article, we have summarized the extensive osseous adaptations required for the obligate bipedal human gait. Below, we focus on the accompanying soft-tissue changes, exploring the consequences of the extended stance on the configuration of the muscles and tendons around the hip, particularly the hip extensors, flexors and abductors. Understanding how these muscles have evolved in the human lineage could be helpful to better understand their injury and overuse patterns.

Fossil preservation of soft tissues is extremely rare, and evolutionary changes of musculature have to be interpreted from the rugosities where muscles are thought to originate. To infer soft-tissue changes, we have to rely more on comparative anatomy than for skeletal changes. Comparative anatomical studies of extant primates allow us to establish form–function relationships, but we should bear in mind that since the last common ancestor of humans and chimpanzees, ∼7 million years ago, both lineages have evolved (though the last common ancestor was more similar to living apes than to living humans).

## ADAPTING TO THE DOUBLE-EXTENSION: MUSCLE ORIGINS, INSERTIONS AND MOMENT ARMS

With increasing fossil evidence, it appears that the suite of adaptations for obligate bipedality evolved in a relatively short time span. One of our early ancestors, *Ardipithecus ramidus* of 4.4 million years old, had a pelvis already much more human- than chimpanzee-like [1]. By default, muscles and their tendons around the hip evolved in synergy with the osseous framework (as outlined in the previous section). As we have seen, the double extension involved ‘compacting’ the pelvis, and development of a long ‘vertical’ femur. Comparing the hip and thigh musculature, the biggest differences between the non-human apes and humans are found in the quadriceps, hamstrings and gluteals. All three muscle groups underwent changes in relative muscle volume in response to the adoption of a habitual bipedal gait. Furthermore, changes in moment arms for the iliopsoas, gluteus maximus and hamstrings occurred, while the gluteus medius and minimus had a shift in their primary function. In comparison, changes for the adductors and hip rotators were slight. For the hip external rotators, such as the piriformis, obturator and gemelli, origin, insertion and function stayed approximately the same during the acquisition of a habitual bipedal gait. This may be explained by the course of the external hip rotators, which, of all hip and thigh muscles, is most parallel to the axis of the double extension, i.e. the femoral neck. The soft-tissue changes summarized earlier limit the power loss that results from moment arm reductions that accompany the double extension, but we will see the hamstrings were likely more affected by the double extension than other muscles.

**Fig. 1.**
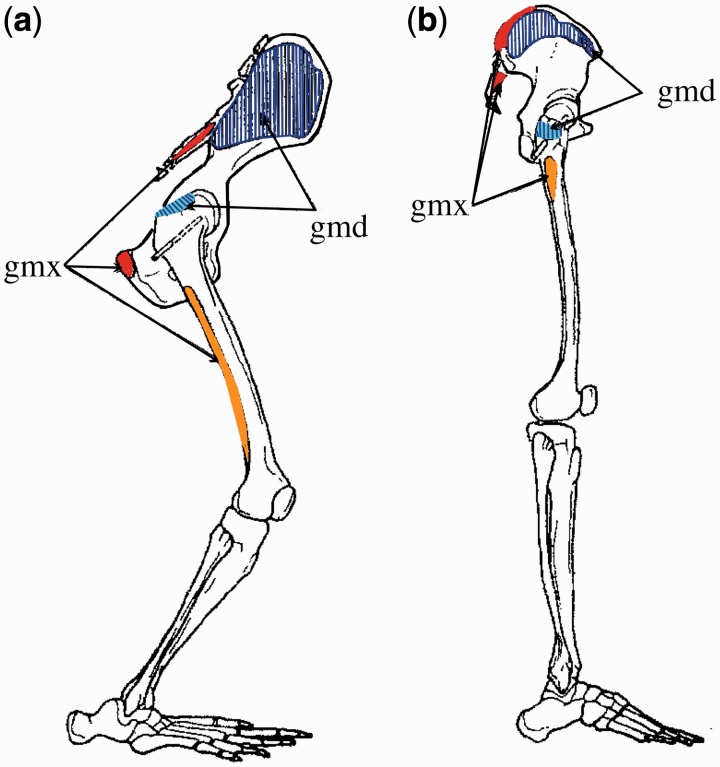
Rearrangement of gluteal origins and insertions. (a) Gorilla and (b) human. Red: gluteus maximus origin, orange insertion. Blue: gluteus medius origin, cyan insertion (adapted from [8], with permission). Gmx, gluteus maximus; gmd, gluteus medius.

In general, the non-human apes have relatively short hindlimbs with powerful muscles (large physiological cross-sectional area). This indicates the importance of generating power over a large range of motion in short limbs for these climbers [2, 3]. In humans, the need for maximum power has shifted to energy efficiency in a smaller range of joint movement with long hindlimbs, suited for running and walking [4, 5]. For the hamstrings and gluteus maximus, this was mainly accomplished by changing their moment arm. But for the gluteus medius and minimus, their primary function changed from hip rotators to true abductors [6] (Fig. 1). The hamstrings and gluteus maximus in chimpanzees, for example, have a relatively long muscle moment arm at the hip and a short lever (i.e. the femur) to move. In addition, the insertion of both gluteus maximus and the medial hamstrings is more distal on the lever they act on, increasing their mechanical advantage [6, 7]. This arrangement confers large extension power to the hindlimbs of the non-human apes, essential for vertical climbing. In humans, the lever for hamstrings and gluteus maximus (the femur) is lengthened, while the muscle moment arms (at hip and/or knee) are shortened, decreasing the power generating capacity of the muscles. The benefit, however, of such rearrangement is that it is suited for generating speed in the form of angular velocity, essential for running.

### Quadriceps

**Fig. 2.**
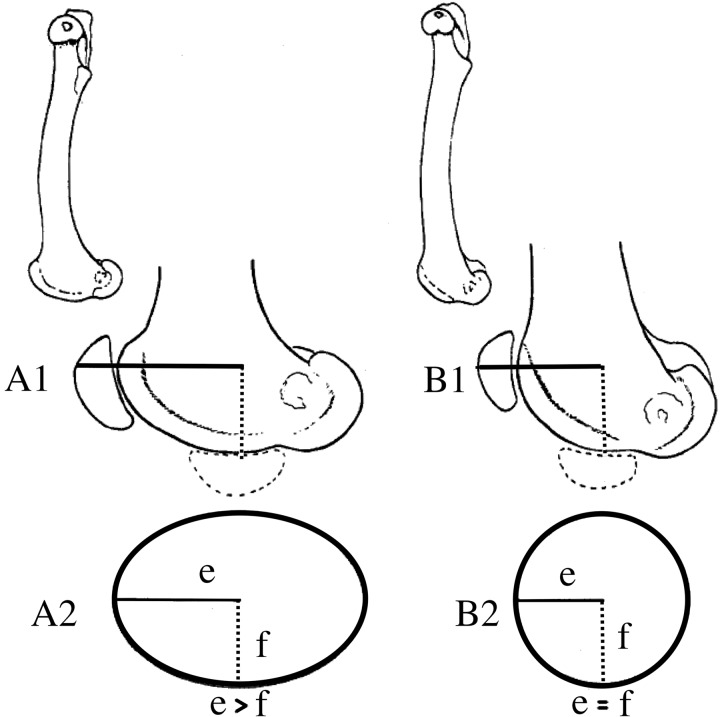
The anteroposterior dimension of the trochlea determines, together with the thickness of the patella, the moment arm of the quadriceps (from [9], with permission). (A) Femur of Bontebok (*Damaliscus dorcas*), a bovid of the open plains. (B) Femur of Bushbuck (*Tragelaphus scriptus*), a bovid of closed forest.

The quadriceps is functionally much more active at the knee than at the hip, only the rectus femoris is bi-articular—spanning both the hip and knee joint—and functions as a relatively weak hip flexor next to being a powerful knee extensor. This is in contrast to the non-human apes who have the highest torque levels at the hip (see part 1). This is an expression of the ‘mid-flex’ default working range of the non-human ape hip versus the extended hip in humans. The mid-flex hip requires powerful hip extensors to counteract flexion torques on a more horizontal femur. While this need for hip extensors is decreased in the extended human hip, torque levels are highest around the knee with activities such as running and stair walking [8]. This helps to explain the relatively large volume of the human quadriceps relative to the hamstrings. A further adaptation that increases quadriceps power is the increase in anteroposterior dimension of the femoral trochlea relative to the femoral condyles. This dimension is increased in sprinters such as antilopes and other bovids (Fig. 2) that rely on top speed versus manoeuvrability of the lower limb [9] and also in bipedal modern humans in comparison to the non-human apes [10].

### Hamstrings

**Fig. 3.**
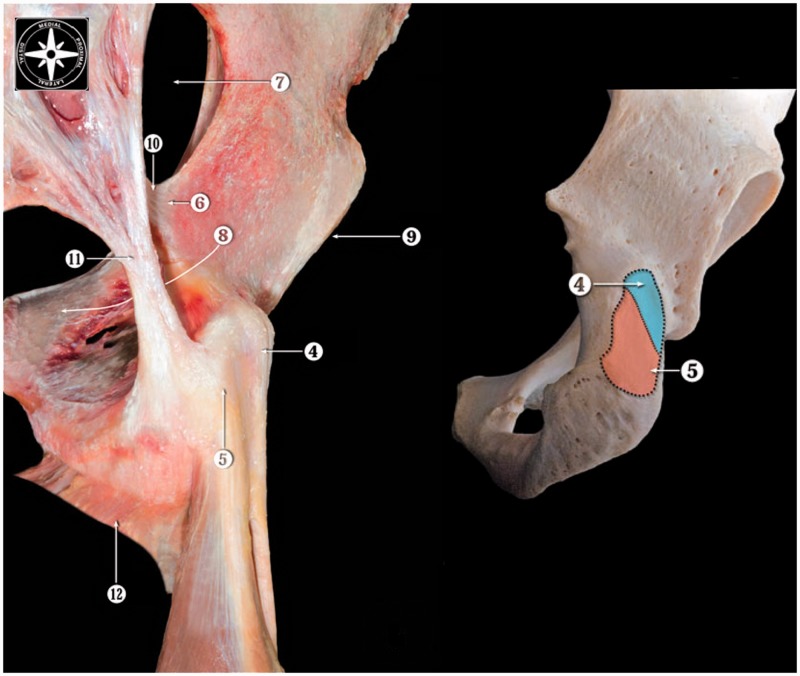
Hamstring origins at the ischial tuberosity. Posterior view on right side of pelvis. The semimembranosus (4) has the position closest to the hip joint (9), and therefore the shortest moment arm. The semitendinosus and biceps femoris share their origin (5), further from the hip joint (9) than the semimembranosus. However, the origin located most distal/dorsal from the hip joint is that of the adductor magnus (yellow oval). Modified from [11], with permission.

The three main components of the hamstrings (sometimes referred to as ‘true hamstrings’) originate from the ischial tuberosity (Fig. 3): the long head of the biceps femoris (inserting laterally on the proximal fibula), the semimembranosus and semitendinosus (inserting medially on the proximal tibia). The short head of the biceps femoris originates from the femur at the linea aspera, and is mono-articular, the true hamstrings are bi-articular, i.e. they are hip extensors and knee flexors.

**Fig. 4.**
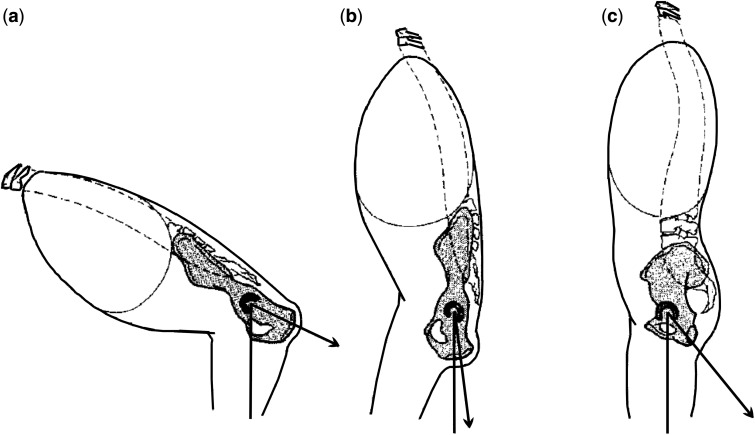
Pelvic lordosis improves the hamstring moment arm in erect humans. (a) Chimpanzee has a long hamstring moment arm (ischium) with a flexed hip, shortening markedly when the hip is extended (b). Pelvic lordosis in humans reduces this shortening (c). From [12], with permission.

**Fig. 5.**
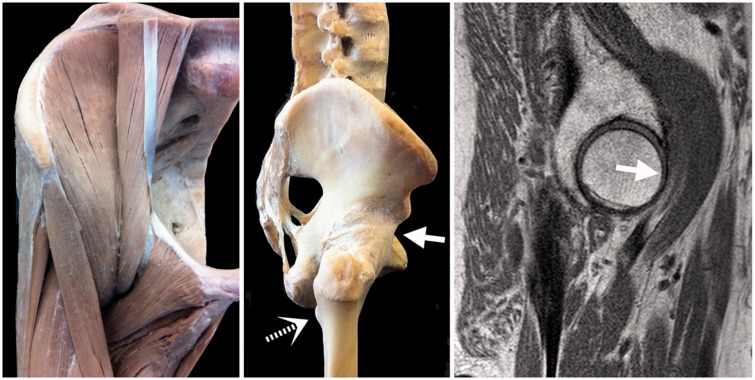
In the double extension, the iliopsoas now has a fulcrum at the femoral head (solid arrow) in its course to the lesser trochanter (dashed arrow). Photographs with permission from the Anatomical Institute, Christian Albrechts University Kiel, Germany.

Compared to humans, chimpanzees have a long ischial tuberosity, which creates a very effective moment arm for the hamstrings in a flexed hip (Figs 1, 4 and 5). The effect of the double extension to the configuration and function of the hamstrings can be illustrated by looking at a hypothetical chimpanzee going from bent-hip bent-knee to an erect posture. Due to its stiff lumbar spine, a chimpanzee can only achieve an erect posture by swinging the pelvis backward over the femoral heads. This brings the origin of the hamstrings, on the ischial tuberosity, near the femur, considerably shortening the hamstring moment arm (Figs 1 and 4). This at once explains why chimpanzees cannot adopt an extended bipedal gait, the leverage of their hamstrings is so unfavourable that the muscles cannot ‘power’ the hindlimb. During bipedalism they will therefore stick to a flexed position of the hip corresponding to a more favourable mechanical advantage of the hamstrings (i.e. 100–120° of flexion during bipedalism [12]). Humans, in contrast, are able to achieve an extended bipedal gait because of the lumbar lordosis which tilts the pelvis forward, reinstating some of the hamstring moment arm. An additional compensation may be that the ischium, nearly parallel to the ilium long axis in the non-human apes, developed a posterior angulation in early humans. This has been termed ‘pelvic lordosis’ and in fact reduces the amount of true lumbar lordosis required to bring the centre of gravity of the trunk over the feet [13]. This posterior angulation brings the ischial origin of the hamstrings to a more dorsal position.

### The gluteals

The mono-articular ‘gluteus maximus’ is a much bigger muscle in erect humans than in the non-human apes because it is not only the strongest hip extensor in modern humans but it also keeps the pelvis (centred) upright over the hips.

In adapting to the double extension, loss of power of the gluteus maximus was compensated by an enlargement of the muscle, especially its cranial portion, and a substantial reorganization of its origin, insertion and, thus, moment arm. In the non-human apes, the gluteus maximus consists of two parts, a proprius and an ischiofemoralis. The gluteus maximus proprius is homologous to the human gluteus maximus; it is a small muscle in the non-human apes, mainly originating from the sacrum and sacroiliac ligament. The ischiofemoralis is located caudally from the gluteus maximus proprius on the ischial tuberosity, is much larger and has a large insertion area, extending far distally on the femur. Its role is comparable to that of the human hamstrings, such as hip extension and deceleration of the swing leg (Lieberman *et al.* [14]). In humans, the ischiofemoralis is absent.

The double extension shortens the moment arm of the gluteus maximus (proprius). This loss was partially compensated by moving the gluteus maximus origin to the ilium (Fig. 1), improving its moment arm when the hip extends beyond the erect position. Thus, the gluteus maximus can (partially) compensate for loss of hamstring power with hip extension. At the femur, loss of the ischiofemoralis part moves the insertion of the gluteus maximus in modern humans to a much smaller and more proximal part of the femoral shaft. This shortens the moment arm of the gluteus maximus, but at the same time generates much more speed (angular velocity) for a given contraction of gluteus maximus [7]. Such speed is important for the shift of walking to running.

Side-to-side balance of the trunk is particularly important during bipedal gait. This balance is for a large part maintained by the ‘gluteus medius’, which is a large and well-developed thigh abductor in modern humans. While the gluteus medius of non-human apes is a medial (internal) rotator of the thigh [15], it can also provide side-to-side balance [6]. This shift in function (but not in role) of the gluteus medius was made possible by the anterior shift of their origins with the flaring of the ilium blades and the ‘verticalization’ of the femur (Figs 1 and 5). Importantly, the proximal femur of humans displays several features that improve the mechanical advantage of the lesser gluteals. The greater trochanter is the insertion site for the gluteus medius and minimus and has a lateral position, which, together with a large neck-shaft angle and relatively long femoral neck, increases the moment arm of the lesser gluteals. At the same time, however, it has a flaring surface which projects medially towards the neck of the femur, thereby lowering the bending moment during abduction. In contrast, the non-human apes have a proximally oriented greater trochanter leading to a small moment arm, yet allowing high hip mobility. The femoral morphology seen in extant non-human apes is considered as a derived morphology to facilitate tree climbing, and is not ancestral (i.e. earlier, or more primitive) to the human proximal femoral morphology.

### The iliopsoas

For the mono-articular ‘iliopsoas’, the strongest hip flexor in modern humans, the situation would appear the same as for the hamstrings: loss of power due to shortening of its moment arm by hip and spine extension. Clearly, loss of moment arm can be (partly) compensated by an increase in muscle volume. But the iliopsoas received an unexpected bonus in that it came to curve around the femoral head with hip extension. The femoral head then works as a fulcrum, increasing iliopsoas flexion power on the femur (Fig. 5). We can appreciate this during surgery: palpate the iliopsoas tendon in a supine patient and feel how tight it runs over the femoral head (Fig. 5) in an extended (neutral) hip. In this neutral hip position, and with further hip extension, the femoral head now works as a pulley for the iliopsoas, increasing its hip flexion moment. Thus, the double extension positioned the iliopsoas ‘around’ the hip, in near-perfect position to help power the forward swing in human walking or running. The iliopsoas is eccentrically loaded during lengthening with the propulsion phase with the foot on the ground. When the foot is lifted, this ‘iliopsoas spring’ is released and the leg swings forward at low energetic cost.

### The external rotators of the hip

The external rotators of the human hip comprise the gemelli, the obturator internus and externus, the quadratus femoris and the piriformis muscle. These short muscles surround the hip joint and are important for hip stability, comparable to the function of the rotator cuff in the shoulder. In the extant non-human apes, however, the obturators show no persistent activity during different postures and gaits, which makes a function as hip stabilizers in these primates unlikely [16].

In humans, the gemelli and obturator internus form a conjoined tendon which inserts on the dorsal half of the medial aspect of the greater trochanter, while the piriformis muscle attaches just posterior and superior to it [17]. The obturator externus originates from the anterior side of the obturator membrane and surrounding pubis and ischium and inserts with a tendon onto the trochanteric fossa. Due to the extended hip position in humans, this tendon can press against the posterior side of the femoral neck where it can demarcate a shallow ‘obturator externus groove’. This groove was suggested as an exclusive trait of the extended human hip posture, but several non-human primates equally possess an obturator groove even though they do not regularly engage in upright gait [18].

In hip preserving surgery, the obturator externus is very useful as a protector of the medial circumflex artery branches that supply the femoral head. During surgical hip dislocation, as long as the obturator externus tendon remains intact, these important vessels are protected from overstretching [19]. Similarly, in the direct anterior approach to the hip, although the conjoined or piriformis tendons may be partially or completely released, preservation of the obturator externus tendon is important to preserve hip joint stability [17].

## RUNNING INTO TROUBLE? MUSCULAR CONSEQUENCES OF THE DOUBLE EXTENSION

The muscular adaptations outlined earlier helped to make modern humans energy-efficient long-distance walkers/runners. However, these adaptations came at the expense of maximum power, characteristic for activities such as tree climbing (in the apes), but equally so for sprinting.

Furthermore, muscle-tendon units have different functions, and this relates to their ‘safety factor’ (strength expressed as failure or yield stress related to a lifetime of loading). In contrast to bone and muscle, where safety factors tend to be uniform between vertebrates of very different size or locomotion type, safety factors differ between tendons in a single animal [20]. This is related to the primary function of a muscle-tendon unit. Their role in storage and release of elastic energy requires that some tendons operate at high stresses (and strains), which compromises their safety factor. Other ‘low stress’ tendons have larger safety factors and primarily function to reduce the amount of stretch their muscles must overcome when contracting to control movement. Thus, tendon elasticity helps muscle fibres contract around their optimal length by dampening length changes of the muscle-tendon unit. Activities such as sprinting may approach or exceed the safety factors of muscle-tendon units that function in elastic energy storage and release.

### Hamstring tears or strains

Hamstring tears or strains are the most frequent sports injuries in the lower leg, ∼4 times more common than quadriceps or calf muscle injuries [21, 22], and ∼6 times more common than ACL tears [23, 24]. The biceps femoris is injured far more often than the medial hamstrings: two magnetic resonance imaging (MRI) studies, including a total of 222 patients, found biceps femoris lesions in 70–80% of hamstring injuries [25, 26].

**Fig. 6.**
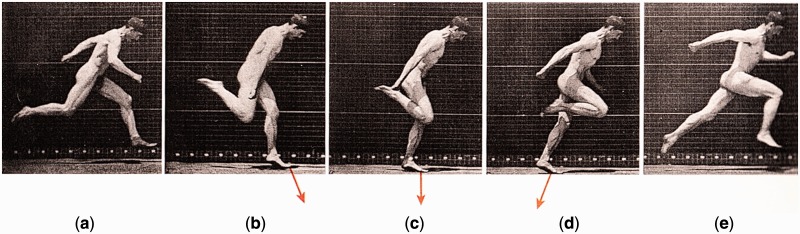
Still images from a running stride as demonstrated by Muybridge in the 1880s. With the pelvis tending to incline forward, maximal hip flexion is ∼90° in sprinting (Fig. 6e). From [31], with permission.

Hamstring muscle tears or strains occur mainly in sports, such as soccer, track and field, rugby, etc., with the most common activity at injury being kicking and sprinting. With increasing running speed, proportionally more work is done by the proximal than distal leg muscles [27], corresponding, not coincidentally, to the architecture of the legs of quadrupedal sprinters, with big muscles proximally, and light bones with long tendons distally [28]. Similarly, with increasing running speed, the leg is used more like a spring with more elastic energy storage and release in tendons. The most common mechanism of injury with sprinting is at terminal swing or early stance phase, when the hip flexes and knee extends simultaneously (Fig. 6a–c) [29, 30]. Terminal swing phase (i.e. a ‘ballistic’ open chain movement) and early stance phase share that the hamstrings are close to their maximum length and that conflicting demands are placed upon them, namely, concentric contraction for hip extension (i.e. muscle fibre shortening) and eccentric contraction for knee extension (i.e. muscle fibre lengthening).

Studies of the last 20 years, as summarized in recent meta-analyses and reviews, have failed to identify causative mechanisms for hamstring injuries [32, 33]. Apart from well-known risk factors such as age and previous hamstring injury, there is currently no conceptual framework for the proposed risk factors or recurrence rate. For example, a meta-analysis of five studies (including in total 216 individuals) examining the difference between hamstrings and quadriceps strength (H:Q ratio) showed that this was not a causative factor for hamstring injuries [33]. Neither have studies measuring dimensions or lengthening of the muscle tendon complex led to conclusive explanations for the high rate of hamstring, and specifically biceps femoris, injuries in sports [11, 34].

Of the hamstrings, particularly the biceps femoris features several characteristics that indicate it functions to store and release elastic energy, requiring its tendons to operate at high stresses (and strains), potentially compromising its safety factor with activities such as sprinting [20].

Compared to human hip flexors and the mono-articular gluteus maximus, the human hamstrings function under unfavourable conditions: they have a relatively low mechanical advantage and no embedded sesamoid bone or other fulcrum. Furthermore, the bi-articular hamstrings have to control both a vertical femur and a long tibia, i.e. long bones that develop high angular velocities and torques. Bi-articular muscles face intriguing tasks, as they serve two joints, and often conflicting demands are placed upon them. During stance phase, for example, the hamstrings both extend the hip and control extension of the knee; the triceps surae performs the same double function in stance phase for knee extension and ankle plantar flexion. This may mean that, at the same moment, the hamstrings absorb energy at the knee and produce energy at the hip. This has been called an ‘energy strap’ and may enhance energy efficiency in running [27], similar to the storage and release of elastic energy in the Achilles tendon–triceps surae mechanism [35]. On the other hand, this may render bi-articular muscles more vulnerable to strain injury.

**Fig. 7.**
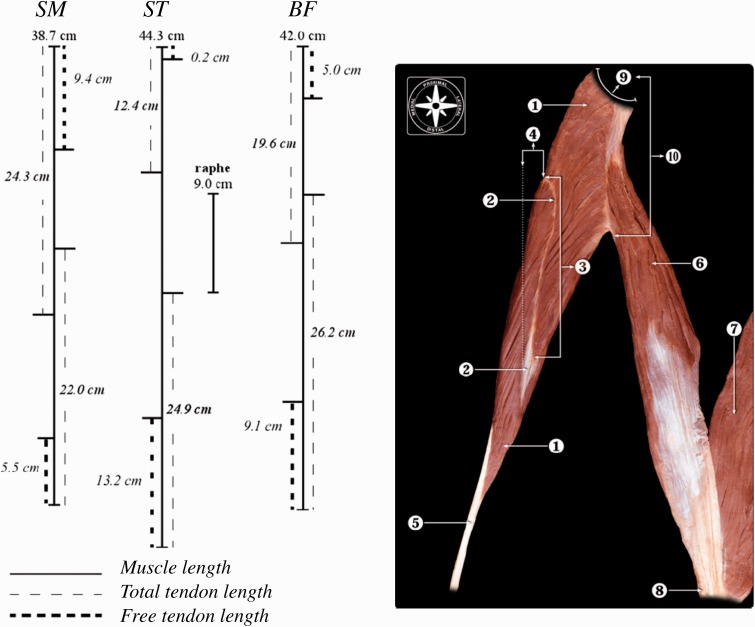
Muscle and tendon lengths of the true hamstrings. The long head of biceps has a long flat tendon with a 2-cm moment arm at the knee, enabling storage and release of elastic energy. 1. Semitendinosus muscle; 2. raphe; 5. semitendinosus tendon; 6. long head of biceps femoris muscle; 7. short head of biceps femoris muscle; 8. biceps femoris tendon; 9. ischial tuberosity; 10. conjoined tendon (long head of biceps femoris and semitendinosus) from [11], with permission.

When we examine the biceps femoris more closely, we find its anatomical aspects may combine to create a function similar to that of the Achilles tendon–triceps surae complex at the ankle. The biceps femoris is a hemi-pennate muscle with relatively short muscle fibres and relatively long tendons at origin and insertion (Fig. 7) [11, 36].

In running, the long Achilles tendon stores and releases elastic energy during stance phase. The efficiency of this stretch and recoil of the Achilles tendon goes a long way to explain the difference in metabolic efficiency between runners [35]. This is because storing and releasing elastic energy costs less energy than muscle contraction. The potential elastic energy of this mechanism increases when the force on the tendon increases. At the ankle joint this means that a shorter calcaneus is more efficient than a long one, because the smaller the moment arm, the more energy is stored in the tendon at given kinematics and kinetics. At the knee joint, this would mean that the biceps femoris tendon is more suited for elastic energy storage and release than the tendons of the medial hamstrings. The biceps femoris has a moment arm of ∼2 cm at the knee, which is nearly constant over the entire knee flexion range. The moment arm of semimembranosus and semitendinosus at the knee is twice as long, and the latter increases further beyond 30° of knee flexion [37]. The relatively short moment arm of the biceps femoris at the knee might give the biceps femoris tendon a higher potential to store and release elastic energy than the medial hamstrings during running. However, this comes at the price of much higher forces in the muscle-tendon unit and may help to explain the much higher injury rate of the biceps femoris compared to the medial hamstrings. As the tendon at origin and insertion will be equally strained, both have a potential to act as elastic springs, hence the moment arm and movement at the hip are equally important (Figs 3 and 7).

## BEYOND THE HAMSTRINGS: BEWARE OF THE DOCTOR

Injuries or overuse of muscle-tendon units around the human hip (other than the hamstrings) are relatively rare, and so are degenerative soft-tissue lesions around the hip. The exception is the gluteus medius which can develop degenerative (partial) tendon tears, either without or with trochanteric pain and gait symptoms. But, other than the hamstring and gluteus medius, most soft-tissue problems around the hip seen in orthopaedic practice may in fact be iatrogenic. Failure to heal of detached tendons, protruding cement or total hip components can cause pain and symptoms related to the lesser gluteals, external rotators and iliopsoas.

### Injury and dysfunction of the gluteals

The human ‘gluteus maximus’ became important to extend the pelvis and spine over the hips, and stabilize it in the sagittal plane. It is particularly important during running [14]. Yet, injuries of the gluteus maximus are exceedingly rare: although it is dutifully listed as a differential diagnosis in posterior pelvic pain, we found not a single (case) report on gluteus maximus tear, strain or overuse. This likely indicates the robustness of the gluteus maximus’ mono-articular architecture and favourable moment arms creating a large safety factor for its use [20], whether in daily activities or peak loading such as in sports.

In strong contrast to the gluteus maximus, ‘gluteus medius’ injuries are relatively common and, unfortunately, many of these are iatrogenic. Abductor weakness with pain and gait disorders can result from the direct lateral (transgluteal) approach to the hip. Dehiscence or denervation of the anterior portion of the gluteus medius after this approach is reported in ∼50% [38] and 10% of patients [39, 40], respectively. To solve the former is very difficult [41–44], to solve the latter impossible. Abductor insufficiency may be further complicated by pre-existing gluteus medius tendon degeneration or tears, which is more prevalent in elderly women [45, 46].

The gluteus medius performs most of the abduction work in bipedal gait of the three abductors, which further include gluteus minimus and tensor fasciae latae (TFL). The gluteus medius is a thick, relatively short muscle, and this architecture is one of the characteristics that signals its primary function is power rather than speed. Due to the length of their muscle moment arm, which is about half that of the moment arm of the ground reaction force (i.e. a mechanical advantage of 0.5), the abductors have to produce a force of approximately twice body weight during monopedal stance during normal gait [47]. When examined in 3D, the TFL has the best line of action for true abduction [48], while the gluteus medius, due to its origin along the entire iliac crest, can be best divided functionally in anterior and posterior portions, active in early and mid to late stance, respectively [49]. The gluteus medius can develop degenerative (partial) tendon tears, either without [50] or with trochanteric pain and gait symptoms [51, 52]. Whether this entity is comparable to degenerative tears of the supraspinatus tendon in the shoulder [53] has not been systematically examined.

A further indication of the high loads on the abductor mechanism at its insertions is that pain around the greater trochanter in the absence of abductor tendon abnormalities is a common entity [54]. This greater trochanteric pain syndrome (GTPS) is seen more frequently with degenerative disorders of the lumbar spine [55] or hip, in women and with obesity, factors that each can increase the required work of the abductors. This is higher in women compared to men, for example, due to a less favourable mechanical advantage of the abductors (ratio of abductor versus body weight moment arm [56]). The pain in GTPS is thought to arise from the tendon insertions or their related bursae [57]. Perhaps, the conspicuous absence of TFL tendon or muscle disorders can be explained by its (bursa-free) insertion on a fascial structure, the iliotibial band, rather than on a bone, which likely decreases peak loads in the TFL muscle-tendon unit.

In the ‘new’ field of soft-tissue endoscopy around the hip, surgeons now perform trochanteric bursectomies, with or without lengthening of the iliotibial band for GTPS. However, none of the small single-surgeon case series reported uses control groups, and no comparison between open and endoscopic surgery has been made [58].

### Injury and dysfunction of the iliopsoas

Again, the most common cause for persistent iliopsoas pain in orthopaedic practice appears iatrogenic rather than overuse or sports [59, 60]. During total hip arthroplasty, insufficient anteversion and/or depth (e.g. in dysplasia) of the implanted cup can cause iliopsoas tendonitis or bursitis where the psoas tendon or iliacus muscle impinges on the cup edge. In contrast to the non-human apes, the double extension led to the development of a psoas groove at the human anterior acetabular rim [61], requiring special attention to seat the cup below this level. Similarly, large-diameter femoral heads may cause iliopsoas impingement, independently of the acetabular component [62].

Iliopsoas impingement with tendonitis/bursitis is characterized by pain with active hip flexion, for example when getting out of a car (‘car sign’ [63]) or walking up a gradient or stairs. However, many patients with hip osteoarthritis (before arthroplasty) have groin pain when testing the iliopsoas. Thus, iliopsoas pain can persist or arise *de novo* after total hip arthroplasty.

When, despite conservative therapy of stretching exercises and steroid injection, these symptoms persist for months, tenotomy of the psoas major tendon is an option. This is usually performed at the insertion at the lesser trochanter, at the head–neck junction or at the anterior acetabular rim. Although continuity of the iliopsoas muscle complex is preserved irrespective of the location of psoas tenotomy [64], MRI studies show atrophy of both the iliacus and psoas major muscles follows and appears permanent [65, 66].

### Injury to the hip external rotators

The external rotators of the hip are detached with the posterior approach to the hip, and whether their repair remains intact postoperatively is reported variably [67–69]. But the external rotators can also be detached during a direct anterior approach in total hip arthroplasty [17], specifically the conjoined (obturator internus and gemelli) and piriformis tendons [70]. Whether reattachment happens after surgery has not been examined for this approach.

## CONCLUSION

The development of human habitual bipedal gait was characterized by compacting the pelvis, double extension of the spine/hip and a vertical, long femur. Accompanying extensive soft-tissue adaptations were changes in relative muscle volume, moment arms and, for some muscles, function. These changes improved energy efficiency of human bipedal walking and (long distance) running, although at the expense of maximum power, characteristic for activities such as tree climbing and sprinting. Spring mechanisms (for storage and release of elastic energy) of the iliopsoas, (lateral) hamstrings and triceps surae help power this efficiency of human walking and running. Strains and overuse injuries of the bi-articular triceps surae and particularly the biceps femoris indicate activities such as sprinting approach or exceed the safety factor of these springs. Conversely, the rarity of iliopsoas strains or overuse injuries may attest for the efficiency of the pulley mechanism at the femoral head and the larger safety factor of mono-articular versus bi-articular muscles. Similarly, the strongest hip extensor, the mono-articular gluteus maximus, is virtually injury-free.

Thus, we find a trade-off between elastic energy efficiency during steady preferred speed running and acceleration as required for sprinting. Tendons for effective elastic energy saving must operate with low safety factors, i.e. they are relatively slender tendons, but for these tendons rapid acceleration is simply not feasible or safe [20]. This corresponds to the non-existence of runners that excel both in sprint and long distance. Modern humans, in a general sense, appear more suited for long distance running than sprinting.

Despite the high loads that occur with locomotion, and excluding the hamstrings and gluteus medius, overuse injuries of muscles and tendons around the hip are quite rare. However, these high loads do create problems with dehiscence after surgical repair of tendons around the hip. Muscle preserving surgical approaches, aiming to avoid tenotomy altogether, are used increasingly, but whether all tendon insertions around the hip are really preserved has to date only been shown for the abductors, not the external rotators [71].

Finally, operations such as trochanteric bursectomy and lengthening of the iliotibial tract are now performed endoscopically. In the field of soft-tissue hip endoscopy, surgeons may learn from their knee and shoulder colleagues. For example, randomized trials (RCT) of operative versus conservative [72], or even sham operation [73] for degenerative meniscus tears show the same results. As further RCTs for acute anterior cruciate ligament tears [74] and subacromial impingement syndrome [75, 76], have also shown equivalent results of operative and conservative treatment, this should drive surgeons to evaluate these new hip endoscopic procedures rigorously.

## CONFLICT OF INTEREST STATEMENT

None declared.
